# Transcranial direct current stimulation (tDCS) over the auditory cortex modulates GABA and glutamate: a 7 T MR-spectroscopy study

**DOI:** 10.1038/s41598-020-77111-0

**Published:** 2020-11-18

**Authors:** K. Heimrath, A. Brechmann, R. Blobel-Lüer, J. Stadler, E. Budinger, Tino Zaehle

**Affiliations:** 1 Department of Neurology, Otto-Von-Guericke University Magdeburg, Leipziger Str. 44, 39120 Magdeburg, Germany; 2 Combinatorial Neuroimaging Core Facility, Leibniz Institute for Neurobiology Magdeburg, Brenneckestr. 6, 39118 Magdeburg, Germany; 3 Center for Behavioral Brain Sciences, Universitätsplatz 2, 39120 Magdeburg, Germany

**Keywords:** Auditory system, Inhibition-excitation balance, Neurophysiology

## Abstract

Transcranial direct current stimulation (tDCS) is one of the most prominent non-invasive electrical brain stimulation method to alter neuronal activity as well as behavioral processes in cognitive and perceptual domains. However, the exact mode of action of tDCS-related cortical alterations is still unclear as the results of tDCS studies often do not comply with the somatic doctrine assuming that anodal tDCS enhances while cathodal tDCS decreases neuronal excitability. Changes in the regional cortical neurotransmitter balance within the stimulated cortex, measured by excitatory and inhibitory neurotransmitter levels, have the potential to provide direct neurochemical underpinnings of tDCS effects. Here we assessed tDCS-induced modulations of the neurotransmitter concentrations in the human auditory cortex (AC) by using magnetic resonance spectroscopy (MRS) at ultra-high-field (7 T). We quantified inhibitory gamma-amino butyric (GABA) concentration and excitatory glutamate (Glu) and compared changes in the relative concentration of GABA to Glu before and after tDCS application. We found that both, anodal and cathodal tDCS significantly increased the relative concentration of GABA to Glu with individual temporal specificity. Our results offer novel insights for a potential neurochemical mechanism that underlies tDCS-induced alterations of AC processing.

## Introduction

Transcranial direct current stimulation (tDCS) is a technique to non-invasively modulate cortical excitability of the human brain by delivering low electrical currents to the cerebral cortex^1,2^. An increasing number of studies demonstrate the potential of tDCS to induce behavioral as well as neurophysiological changes in various cognitive and perceptual domains^3–8^. While tDCS is applied in several interventional and research approaches, the knowledge about the underlying neurophysiological mechanisms is still not complete^9^. By applying tDCS over the motor cortex, the seminal work of Nitsche and Paulus (2000, 2001) demonstrated polarity-specific changes of cortical excitability ^10,11^. The somatic doctrine deduced therefrom assumes that anodal tDCS typically increases and cathodal tDCS decreases cortical excitability in the region under the electrode by shifting the resting membrane potential^1,12,13^. In addition, tDCS aftereffects may outlast the stimulation period by several minutes or hours^11,12,14,15^. While anodal aftereffects have been reported to induce long-term potentiation (LTP) due to enhanced firing rate, cathodal tDCS is supposed to reduce firing rate followed by long-term depression (LTD)^15–18^. However, the somatic doctrine is debated, as dual-polarity effects have mainly been demonstrated in the motor domain and are not commonly transferable on cognitive and perceptual functions^19^. Particularly in the auditory domain, opposing effects regarding the somatic doctrine have been reported. More specifically, electrophysiological data demonstrate an opposite anodal/cathodal dichotomy, with e.g., decreased cortical reactivity of specific brain regions after anodal^20,21^ and increased cortical reactivity after cathodal stimulation^22^. Different stimulation parameter such as stimulation power, electrode size, and electrode placement as well as individual auditory stimuli and the kind of task under investigation have been assumed to contribute to these varying tDCS-effects^9^.

On the neurochemical level, the regional cortical neurotransmitter balance in the electrical stimulated cortex, measured by glutamate (Glu) and gamma-amino butyric acid (GABA) concentrations, provides a meaningful interpretation of the outcome variations. GABA is the major inhibitory neurotransmitter in the brain and specifically in the central auditory system^23^, whereas Glu is the primary excitatory brain neurotransmitter^24–26^. More importantly, activity-dependent synaptic plasticity such as LTP and LTD depends on modulation mediated by both glutamatergic and GABAergic interneurons^27–29^. Pharmacological investigations emphasize the involvement of GABAergic^30,31^ and glutamatergic activity^16,30,32^ shaping tDCS-induced effects. Assuming that tDCS-related alterations of the neurotransmitter level may affect homeostatic plasticity in the stimulated area, Krause et al. (2013) captured a mechanism of neurotransmission function by taking into account the relative concentration of GABA to Glu as an overall index for cortical excitability. They proposed that only a balanced GABA to Glu ratio leads to an optimal level of processing efficiency. Anodal and cathodal tDCS may differentially shift the GABA/Glu balance and thus induces altered homeostatic plasticity.

The use of proton magnetic resonance spectroscopy (H-MRS) allows measuring in vivo changes in cortical neurotransmitter concentrations within a defined region of interest. Indeed, in healthy subjects anodal tDCS over the motor cortex reduces local GABA^34–39^, whereas Glu is reduced after cathodal tDCS^37^. Notably, several further studies that investigated the GABA and Glu system provided heterogeneous results with opposite effects for stimulation protocols of the parietal cortex^40^ or no effects after prefrontal cortex stimulation^41,42^. To what extent tDCS influences local GABA and Glu in primary sensory cortices—and in particular in the human auditory cortex (AC)—remains elusive. Only one study assessed the influence of anodal tDCS over the posterior superior temporal gyrus (pSTG) and demonstrated no significant changes of GABA and Glu in combination with glutamine (Gln) using 3 T MRS^43^. However, low-magnetic field MRS has only been proven to reliably detect metabolites such as N-acetyl-aspartate (NAA), creatine, and choline, but not GABA and Glu. Accordingly, low-field strengths below 3 T MRS have technical limitations in separating Glu and Gln along their respective multiplets and in detecting GABA by overlaying signals^44^. Due to an increased sensitivity and chemical shift dispersion, high-field 7 T MRS provides spectra with high signal-to-noise ratio (SNR) and a spectral resolution for accurate metabolite quantification^45^. Accordingly, the acquisition of spectroscopic data with a magnetic field strength of 7 T provides more detailed information of the underlying changes of GABA and Glu. In the AC, tDCS induced changes on neurotransmitter levels measured by ultra-high-field MRS have not been investigated so far. Therefore, we investigated alterations of the relative concentration of GABA to Glu in the AC before and after auditory tDCS in healthy subjects by means of 7 T MRS. Based on the proposed concept by Krause et al. (2013), we hypothesized polarity-dependent tDCS alterations of the GABA/Glu ratio.

## Materials and methods

### Participants

25 male subjects (mean age 28.76 years, SD 5.81 years, range 20–40 years) participated in this study. We choose only male subjects as the menstrual cycle significantly influences the GABA concentration^46,47^. Subjects gave written informed consent in accordance with the 2013 World Medical Association Declaration of Helsinki. All subjects were native German speakers, and had no history of neurological, psychological or hearing impairment. All procedures were approved by the Ethics Committee of the University of Magdeburg (Germany).

### Transcranial direct current stimulation

All participants received anodal and cathodal stimulation in two separate sessions over the left or right AC. The reference electrode was placed over the contralateral AC. The order of the stimulation sessions was counterbalanced across subjects. The sessions were separated by at least 24 h to avoid carry over effects. TDCS was applied by a battery driven constant current stimulator (DC-Stimulator, NeuroConn GmbH, Germany) using two rubber electrodes placed in 0.9% salinesoaked synthetic sponges. Two 5 × 7 cm stimulation electrodes were placed over T7 and T8 according to the 10–20 system for EEG electrode placement. The stimulation electrode placement has been shown to modulate central auditory processing and cortical reactivity in the AC^22,48–50^. The direct current was applied for 30 min with a strength of 1.5 mA and 10 s fade in/out.

### MRS acquisition

All measurements were performed using a 7 T MR scanner with a 32-channel head array coil (Siemens Healthineers, Erlangen, Germany). High-resolution anatomical images were acquired using the three dimension (3D) magnetization-prepared rapid gradient echo (MPRAGE) sequence with the following parameters: echo time (TE) = 2.73 ms, repetition time (TR) = 2300 ms, inversion time (TI) = 1050 ms, flip angle = 5°, bandwidth = 150 Hz/pixel, acquisition matrix = 320 × 320 × 224, isometric voxel size = 0.8 mm^3^. We applied a stimulated-echo acquisition mode (STEAM) sequence using short TE/mixing time (TM; 20 ms/10 ms) and variable-rate selective excitation (VERSE) radio frequency (RF) pulses. The VERSE pulses were applied because they significantly improved metabolite detection with a short TE/TM STEAM sequence when taking the reduction of the peak power requirements of RF pulses into account^51^. Spectroscopy voxel with a voxel size of 2 cm × 1.5 cm × 1 cm = 3 cm^3^ was manually placed in the AC region. In each MRS-tDCS session, MRS spectra were acquired over three baseline time points and four time points post-tDCS. The three baseline measurements were performed before tDCS sampled at every ~ 10 min interval for approximately 30 min (pre-tDCS measurements). After pre-tDCS measurements, the subjects were removed from the scanner for mounting tDCS electrodes over the left and right AC and tDCS was applied (see above). Subsequently, electrodes were removed, the subjects were re-entered into the scanner and four consecutive post-tDCS measurements followed. Electrode removal from the head, participant table docking in the scanner, running localizer, voxel placement, and shimming were performed between the end of the tDCS and the start of the first post-tDCS measurement. The time interval between the end of the tDCS and the start of the first post-stimulation measurements was on average 14.16 min ± 1.42 min. The four consecutive poststimulation measurements were sampled at every ~ 10 min interval for approximately 40 min (post-tDCS measurements). Subjects were instructed and video-monitored to stay awake during MRS acquisition. To ensure identical positions in all four post-tDCS measurements, MRS voxels were automatically positioned using a vendor-provided automatic voxel positioning (AutoAlign) technique, and were manually placed by an experienced technician^52^.

### MRS data analysis

All spectra were analyzed using LCModel version 6.1.0^53^. We pooled all spectra of the left and right AC separately for the anodal and cathodal stimulation condition. Metabolite concentrations were expressed using institutional units (i.u.). Standard criteria were applied for excluding spectra with poor quality: (i) CRLB of GABA and Glu > 20%, (ii) FHWM > 25 Hz, and (iii) SNR < 8. The concentration of GABA and Glu was corrected for the proportion of grey matter within the voxel^37,54,55^. The group wise outliers of GABA and Glu concentrations, defined as greater than three times the interquartile range, from the remaining spectra were further detected using Boxplot in SPSS 24 (for Windows) and were removed. Missing concentration values due to the exclusion of individual spectra with dissatisfactory quality or classified outliers were replaced by mean levels of each metabolite concentration and each time point. In total, four subjects were excluded. Spectra of three subjects were discarded due to dissatisfactory quality for analysis and one subject did not attend the second session. As an index for cortical excitability, we calculated the ratio of GABA to Glu.

## Results

To test for potential baseline variations for GABA, Glu, and the GABA/Glu ratio across the three pre-stimulation measurements, we first performed a repeated measures analysis of variance (RM-ANOVA) with the within-subject factors *stimulation condition* (anodal, cathodal) and *baseline time point* (pre-tDCS 1, pre-tDCS 2, pre-tDCS 3). The analyses revealed no significant main effect of the factor *baseline time point* for GABA (F (2, 40) = 0.277, p = 0.76, η^2^_p_ = 0.014), Glu (F (2, 40) = 1.427, p = 0.25, η^2^_p_ = 0.067) nor for the GABA/Glu ratio (F (2, 40) = 0.18, p = 0.84, η^2^_p_ = 0.01). Furthermore, RM-ANOVA revealed neither significant main effects of the factor *stimulation condition* nor significant interactions of *baseline time point *× *stimulation condition *(*all p* > 0.1). Accordingly*,* neurotransmitter levels of the three baseline measurements were averaged for further analyses.

**Figure 1:**
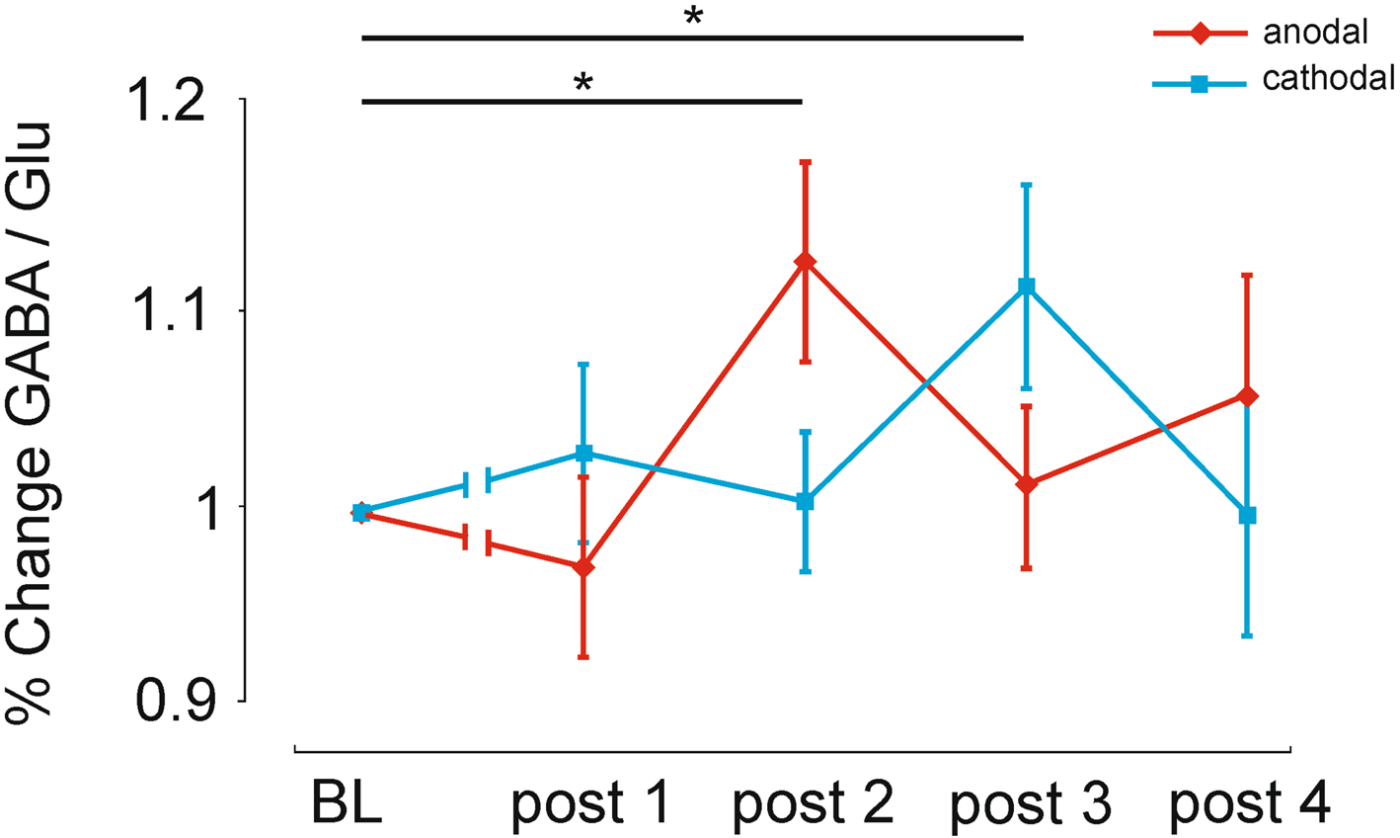
Mean GABA/Glu ratio after anodal and cathodal stimulation. The graph shows a significant increase of GABA/Glu ratio after anodal stimulation during the second post-tDCS measurements and a significant increase of GABA/Glu ratio after cathodal stimulation during the third post-tDCS measurements with respect to the baseline condition (*p < 0.05). Error bars reflect standard error of the mean. N = 21.

To investigate tDCS effects on neurotransmitter level changes, RM-ANOVA was performed with the within-subject factors *stimulation condition* (anodal vs. cathodal) and *time point* [baseline, post-tDCS measurement (1–4)] for the GABA/Glu ratio. Additionally, subsequent exploratory analyses separately for GABA and Glu relative to baseline were performed. One-sample *t* tests (confidence interval 95%) were conducted as post-hoc analyses. For the GABA/Glu ratio, the 2 × 5 RM-ANOVA revealed a statistical trend for the factor *stimulation condition* (F (1, 20) = 3.581, p = 0.07, η^2^_p_ = 0.152), no significant effect of the factor *time point* (F (4, 80) = 0.907, p = 0.46, η^2^_p_ = 0.043), but a significant *stimulation condition *× *time point* interaction (F (4, 80) = 2.555, p = 0.04, η^2^_p_ = 0.113). For illustration, the data were baseline normalized (cf. Fig. 1).

After anodal tDCS, the GABA/Glu ratio remained at baseline level during the first post-tDCS interval (t(20) = 0.562, p = 0.58) followed by a significant increase during the second post-tDCS interval (t(20) = 2.288, p = 0.03). Thereafter, GABA/Glu ratio returned to baseline level at the third (t(20) = 0.332, p = 0.74) and fourth post-interval (t(20) = 0.86, p = 0.4). For cathodal tDCS-condition, post-hoc analysis showed no significant changes at the first (t(20) = 0.607, p = 0.55) and second post-tDCS measurements (t(20) = 0.073, p = 0.94). Thereafter, the GABA/Glu ratio significantly increased (t(20) = 2.101, p = 0.05) at the third post-tDCS interval, before returning to baseline level (t(20) = 0.032, p = 0.97).

**Figure 2:**
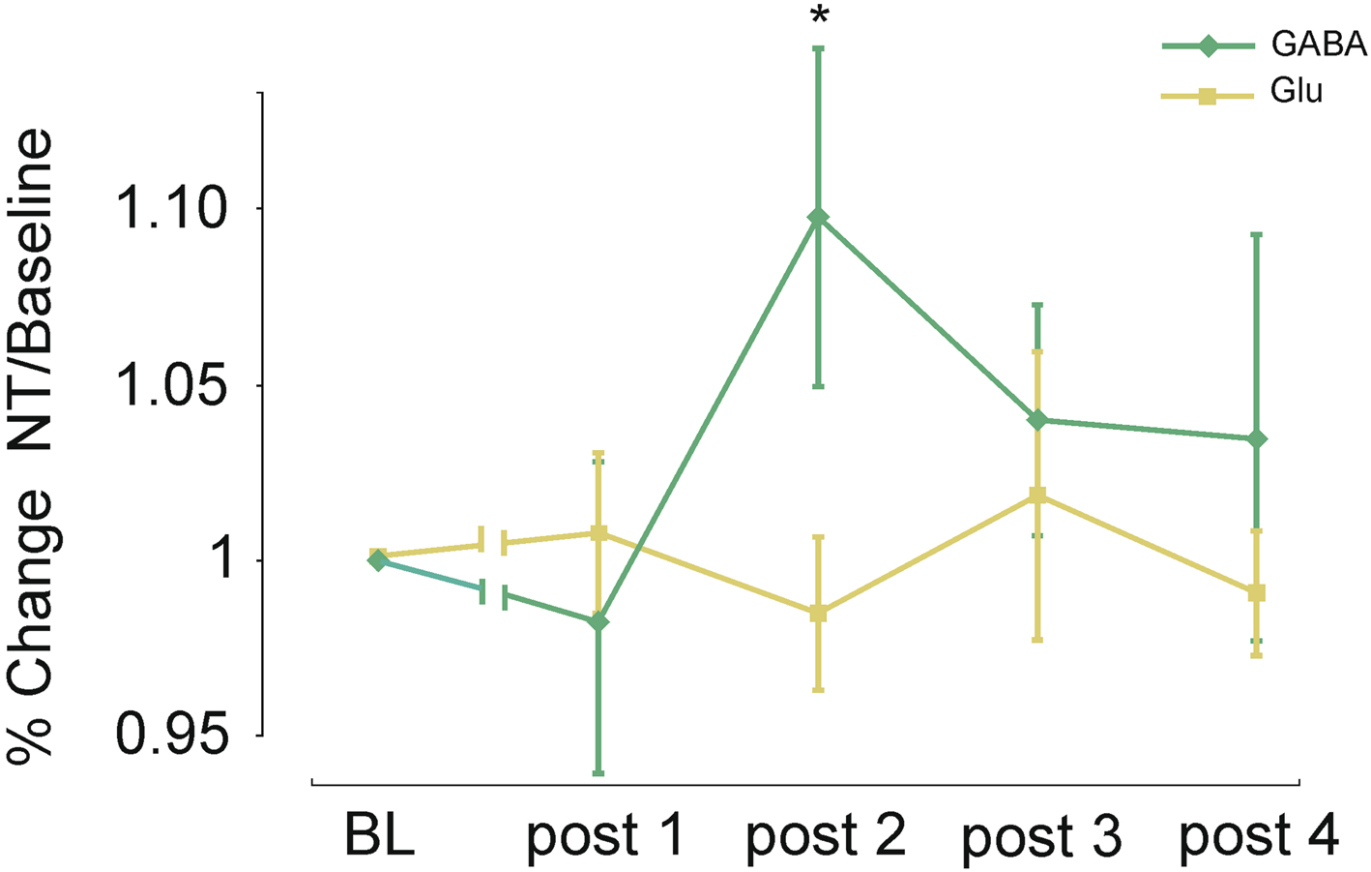
Mean neurotransmitter (NT) concentration after anodal stimulation. Graph shows a significant increase of GABA concentration with respect to the Glu concentration during the second post-tDCS measurements after anodal stimulation (*p < 0.05). RM-ANOVA revealed no significant *neurotransmitter *× *time point* interaction (p = 0.12). Error bars reflect standard error of the mean. N = 21.

**Figure 3:**
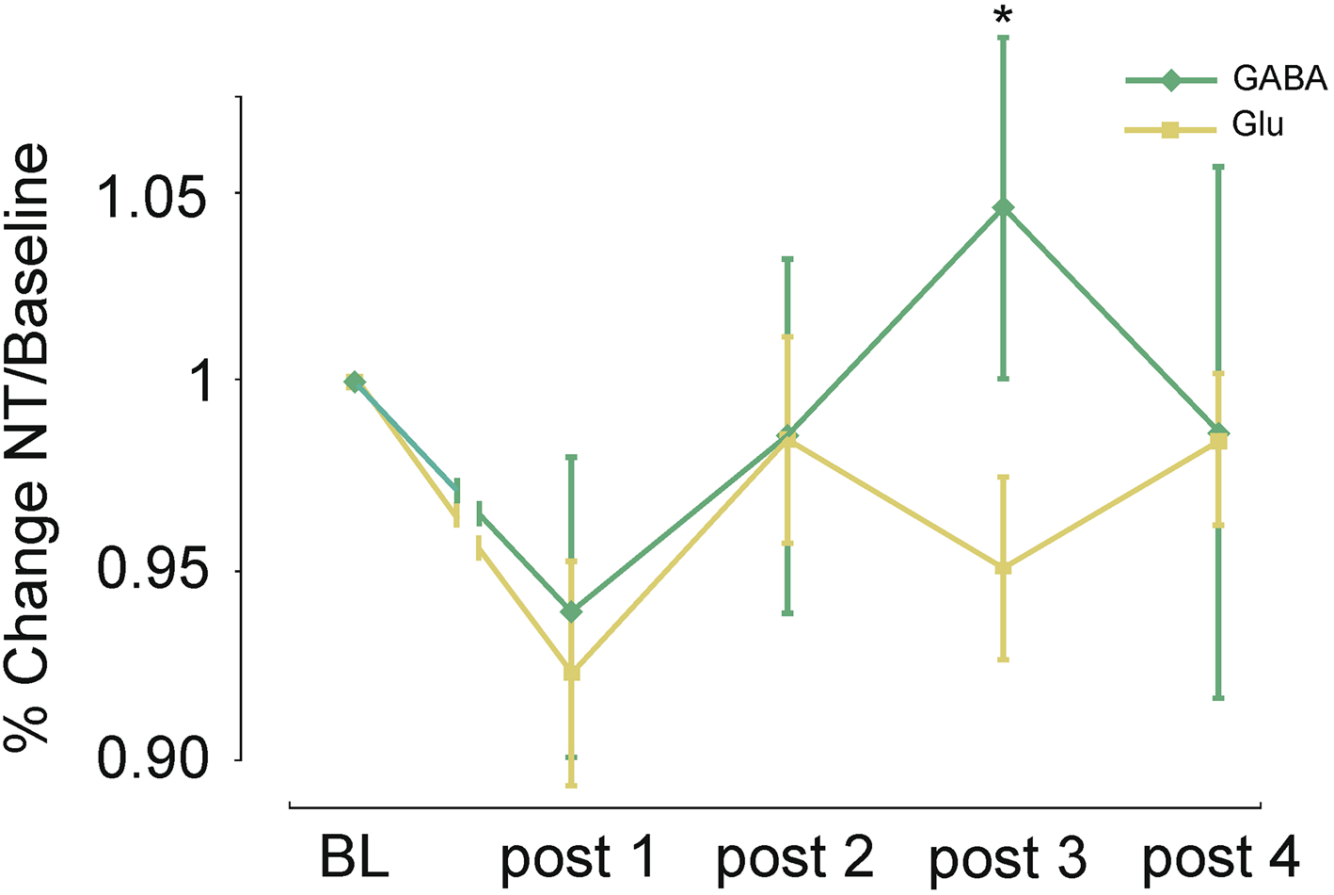
Mean neurotransmitter (NT) concentration after cathodal stimulation. Graph shows a significant increase of GABA concentration with respect to the Glu concentration during the third post-tDCS measurements after cathodal stimulation (*p < 0.05). RM-ANOVA revealed a non-significant trend for *neurotransmitter *× *time point* interaction only (p = 0.06). Error bars reflect standard error of the mean. N = 21.

To further elucidate these alterations of the GABA/Glu ratio during the critical post-tDCS intervals, two-sample *t* test analysis of baseline-normalized data was performed directly comparing relative changes in GABA and Glu separately for anodal and cathodal tDCS. For the anodal condition at post-tDCS interval 2, the analysis demonstrates an increment of GABA concentration relative to Glu concentration (t(20) = 2.206; p = 0.04) (cf. Fig. 2). For the cathodal condition at post-tDCS interval 3, the analysis revealed statistically increased GABA concentration relative to Glu concentration (t(20) = 2.108; p = 0.05) (cf. Fig. 3).

Subsequently changes of GABA and Glu concentrations in response to tDCS were analyzed by RM-ANOVAs with the within-subject factors *neurotransmitter* (GABA vs. Glu) and *time point* [baseline, post-tDCS measurement (1–4)] separately for anodal and cathodal tDCS. For anodal tDCS the 2 × 5 repeated-measures ANOVA revealed a significant main effect of the factor *neurotransmitter* (F (1, 20) = 555, p < 0.001, η^2^_p_ = 0.97), but neither a significant main effect of the factor *time point* (F (4, 80) = 0.537, p = 0.71, η^2^_p_ = 0.026) nor a significant interaction of *neurotransmitter *× *time point* (F (4, 80) = 1.819, p = 0.13, η^2^_p_ = 0.083) (cf. Fig. 2). For cathodal tDCS the 2 × 5 RM-ANOVA revealed a significant main effect of the factor *neurotransmitter* (F (1, 20) = 722, p < 0.001, η^2^_p_ = 0.97), no significant main effect of the factor *time point* (F (4, 80) = 1.78, p = 0.14, η^2^_p_ = 0.082), but a statistical trend for *neurotransmitter *× *time point* interaction (F (4, 80) = 2.307, p = 0.06, η^2^_p_ = 0.103) (cf. Fig. 3).

In sum, while both anodal and cathodal tDCS not directly affected single neurotransmitter concentration, they both modulated the GABA/Glu ratio with an individual temporal characteristic showing a different velocity of the modulatory effect.

## Discussion

In the present study, we examined tDCS-induced changes of neurotransmitter concentration in the AC of healthy adult subjects by using 7 T MRS. We hypothesized that anodal and cathodal tDCS of the AC will systematically alter the ratio of GABA to Glu.

Our findings suggest that the extent to which tDCS modulates behavior is not associated with a single neurotransmitter, but instead relates to the fine-tuned balance of excitation and inhibition as proposed by Krause et al. (2013). Both, anodal and cathodal tDCS significantly changed the ratio of inhibitory GABA to excitatory Glu with individual temporal specificity. Anodal tDCS increased GABA to Glu ratio approx. 25–35 min after the end of a 30 min stimulation (second post-tDCS interval), whereas cathodal tDCS increased the GABA to Glu ratio approx. 35–45 min after the end of stimulation (third post-tDCS interval). To our knowledge, this is the first ultra-high-field MRS study providing evidence for tDCS-induced neurochemical effects in the AC of healthy subjects. In particular, the present results give new insights into the time course of tDCS acting on GABA and Glu concentrations.

Previous studies suggest that changes of GABA and Glu levels in the motor cortex may vary over a time period of 90 min after stimulation^34,36,37,54^. Moreover, electrophysiological studies demonstrated tDCS-induced aftereffects that outlast the stimulation period by several minutes or hours^11,12,14,15^. Such aftereffects have been found to be associated with LTP or LTD that depends on glutamatergic and GABAergic interneurons^27–29^. However, the precise temporal nature of changes of GABA and Glu concentrations in response to tDCS is still unclear and requires further studies.

Yet, it still remains the question about the neurophysiological mechanism of tDCS effects, as neurotransmitter are phasically (cytoplasmic) and tonically (extracellular, vesicular) active^56,57^. Given that MRS is not sensitive to differentiate between these sources, we can only speculate about the putative mechanisms that change the ratio of GABA to Glu concentration. Despite tDCS did not significantly modulate GABA or Glu separately, we found systematic modulations of the GABA/Glu ratio after both, anodal and cathodal tDCS. It is well documented that GABA and Glu interact in the same biochemically pathway, because Glu is the primary precursor for GABA synthesis^24,58^. Since GABA is synthesized from the alpha decarboxylation of Glu by glutamic acid decarboxylase (GAD-67), the activity-driven expression of GAD-67 critically controls GABA synthesis and determines the concentration of GABA in interneurons^59^. In a healthy and optimally functioning nervous system, anodal and cathodal tDCS might subtly interfere with GAD-67 activity and consequently with this pathway resulting in a change of the GABA/Glu ratio. This molecular mechanisms provides a reasonable explanation for tDCS action, however, the exact neurobiological framework is unclear and needs to be addressed in further studies.

The majority of studies on tDCS-induced changes with respect to GABA and Glu were performed by 3 T MRI scanner and rather provide heterogeneous results that vary with the targeted cortical area^19,60^. While a number of studies reported the absence of tDCS induced changes of GABA or Glu level in the prefrontal^42,61^ and motor cortex^55,62^, Clark et al. (2011), for example, reported enhanced Glu in combination with Gln concentration after anodal tDCS over the parietal cortex. Such dissimilar outcomes may also be explained by methodological differences of MRS data acquisition by low-magnetic field strength compared to high-magnetic field strength measurements. 3 T MRS has lower signal dispersion, thus, the detection of GABA and Glu can be challenging due to low concentration and signal overlap with more concentrated metabolites like NAA or creatine^63^. Similar to our voxel position, one study measured neurochemicals in the pSTG by 3 T MRS, with the result of no significant differences in neurotransmitter concentrations between anodal and sham tDCS^43^. Ultra-high field MRS, as used in our study, has the advantage of increased spectral resolution and SNR compared to low-field MRS^64–66^. We are confident that the acquisition of spectroscopic data before, during, and after the application of auditory tDCS will essentially help to assess the induced changes in neurotransmission and thereby to improve the efficiency of auditory tDCS schemes in therapeutic approaches of developmental language impairments. It has been shown that the maturational adjustment of excitatory and inhibitory neurotransmitters is critical for developmental reading ability and might be distorted in children with dyslexia^67,68^. Therefore, the tDCS-induced optimization of GABA and Glu levels and the consequent fine-tuning of cortical excitation and inhibition may lead to “regular” auditory processing^33^.

There are some questions raised by the present data that should be addressed in further investigations. First, in the present study we assessed—for the first time—tDCS induced neurotransmitter changes in the human primary AC. As a consequence, we assessed the effects of both, anodal and cathodal tDCS on the primary AC and compared it to a repeated baseline measure. Even though there is ample evidence for a stable GABA concentration^53^ or even a trend for a decrease in GABA and an increase in Glx during rest^69^, we cannot completely rule out potential changes of neurochemical concentrations over time. Second, our observation of an polarity independent, but asynchrone effect of the GABA/Glu ratio might be related to a functional regulation of Glutamic acid decarboxylase (GAD-67). GAD converts glutamate into GABA and is a key enzyme in the dynamic regulation of neural network excitability. So far, the exact mechanisms underlying polarity dependent tDCS effects on GABA and Glu synthetisation are not yet fully identified. It was found that anodal tDCS reduces GAD-67, while cathodal tDCS reduces the Glu-synthesizing enzyme glutaminase (GLS)^70^. Finally, our data seem to contradict the general assumption that anodal and cathodal tDCS produce LTP and LTD-like effects. It has been shown that anodal DCS can induce LTP^71–73^ whereas cathodal tDCS reduced it^74^. However, LTP enhancement and LTD reduction have been also reported after both, anodal as well as cathodal stimulation^75^. Here the effect depended on the dendritic location; while cathodal DCS enhanced LTP in apical dendrites, anodal DCS enhanced LTP in basal dendrites. Both anodal and cathodal tDCS reduced LTD in apical dendrites. Therefore, future studies are needed to clarify the effects of tDCS on GAD and LTP/LTD in the primary AC.

## Conclusion

To the best of our knowledge, this is the first study that investigated tDCS effects on neurochemicals in the human AC. Our results demonstrate that both, anodal and cathodal tDCS over the AC can alter the concentration of the ratio GABA to Glu within this cortical region. This study extends the view of tDCS-related neurophysiological changes in the AC and encourages further research aimed at understanding neurochemical changes associated with behavioral outcomes.
